# Mini-review of clinical data service platforms in the era of artificial intelligence: A case study of the iHi data platform

**DOI:** 10.37796/2211-8039.1643

**Published:** 2025-03-01

**Authors:** Yu-Ting Lin, Ya-Chi Lin, Hung-Lin Chen, Che-Chen Lin, Min-Yen Wu, Sheng-Hsuan Chen, Zi-Han Lin, Yi-Ching Chang, Chuan-Hu Sun, Sheng-Ya Lu, Min-Yu Chiang, Hui-Chao Tsai, Mei-Ju Shih, David Ray Chang, Fuu-Jen Tsai, Hsiu-Yin Chiang, Chin-Chi Kuo

**Affiliations:** a Big Data Center, China Medical University Hospital, China Medical University, Taichung, Taiwan; b Department of Biomedical Informatics, China Medical University, Taichung, Taiwan; c Division of Nephrology, Department of Internal Medicine, China Medical University Hospital and College of Medicine, China Medical University, Taichung, Taiwan; d Department of Biomedical Informatics, Harvard Medical School, Boston, MA, USA; e Department of Medical Research, China Medical University Hospital, China Medical University, Taichung, Taiwan

**Keywords:** Artificial intelligence, Data ecosystem, Data platforms, Digital twin, iHi platform

## Abstract

In the past two decades, healthcare organizations have transitioned from the early stages of digitization and digitalization to a more comprehensive process of digital transformation, a shift significantly accelerated by the advent of artificial intelligence (AI). Consequently, the development of high-quality clinical data warehouses, derived from electronic health records (EHRs) and enriched with multidomain data, such as genomics, proteomics, and Internet of Things (IoT) information, has become essential for the creation of the modern patient digital twin (PDT). This approach is critical for leveraging AI in the evolving landscape of clinical practice. Leading medical centers and healthcare institutions have adopted this model, as summarized in this review.

Since 2020, China Medical University Hospital (CMUH) has been constructing its data ecosystem by integrating EHRs with extensive genomic databases. This initiative has led to the development of a data service platform, the ignite Hyper-intelligence (iHi®) platform. The iHi platform serves as a case study exemplifying the workflow of the smart data chip, which facilitates the deep cleaning and reliable de-identification of clinical data while incorporating analytical platforms related to genomics and the microbiome to enhance insight extraction processes. The ability to predict complex interactions and disease trajectories among PDTs, digital counterparts of healthcare professionals, and virtual socioeconomic environments will be pivotal in advancing personalized healthcare and optimizing patient outcomes. Future challenges will involve the unification of cross-institutional data platforms and ensuring the interoperability of AI inferences—key factors that will define the next era of AI-driven healthcare.

## 1. Introduction

Healthcare data serves as the cornerstone of artificial intelligence (AI) development in the modern era [1–3]. However, the prevalence of fragmented healthcare data silos—an inherent limitation of contemporary electronic health records (EHRs)—poses significant challenges. These silos contribute to the formation of clinical AI service barriers, impeding the development of a sustainable AI service ecosystem and complicating the creation of an integrated AI landscape. Despite the importance of addressing this issue, systematic evaluations of healthcare data governance and the quality of data service ecosystems, particularly in terms of clinical applications and enterprise adoption, remain scarce [4]. While several leading medical centers have developed proprietary data ecosystems to support advancements in clinical AI, none have tackled the resolution of fragmented AI deployment within the framework of data intelligence. As a result, AI silos persist, leading to considerable inefficiencies in research and development, which often fail to inform policy-making or drive transformations in care models [5].

**Fig. 1:**
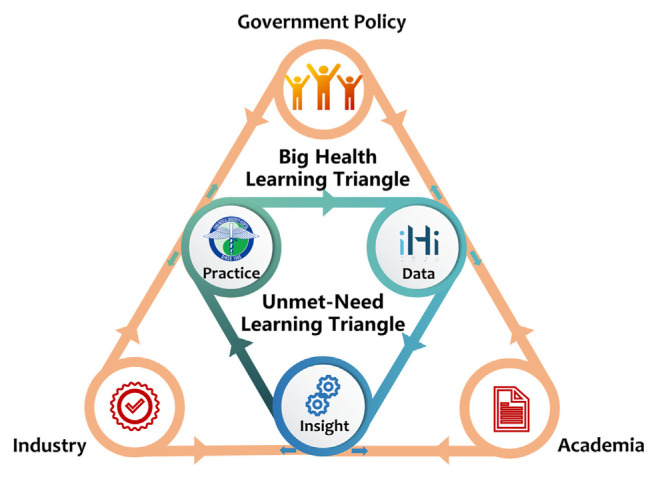
The Dual-Triangle Model of the iHi Data Platform: Enhancing AI Sustainability in Healthcare. The inner triangle illustrates the core components—Data, Insight, and Practice— highlighting the transformation of data into actionable insights and their subsequent application in clinical practice. This foundational structure underpins the broader objectives represented by the outer triangle, which encompasses the needs of Government, Academia, and Industry. Together, these interconnected elements enable the platform to generate innovative insights, address the dynamic requirements of healthcare, and foster cross-sector collaboration.

In response to these challenges, the Big Data Center (BDC) of China Medical University Hospital (CMUH) launched the ignite Hyper-intelligence (iHi®) platform in 2020. This initiative aims to create a solid infrastructure for the development and validation of healthcare AI technologies, while facilitating improved data-human interaction and the integration of AI solutions into daily practice. In addressing the common issue of “data hunger” in the healthcare AI development ecosystem, the platform focuses on the automatic preparation of high-quality datasets for healthcare professionals, thus efficiently meeting critical needs. By establishing a “health learning triangle” that emphasizes the transition from data to actionable insights and, ultimately, to clinical practice, the iHi platform enhances the sustainability of the AI ecosystem across the healthcare sector. This model fosters a unique AI ecosystem learning triangle, linking the healthcare industry, policymaking, and scientific discovery (Fig. 1).

This review examines recent advancements in modern data service platforms and evaluates how their data strategies align with our double-triangle data learning model. The primary aim of this article is to explore the potential for global unification of data service platforms, a critical step toward dismantling the persistent cycle of AI service silos in clinical practice. This, in turn, would support the transition toward a global digital twin initiative, paving the way for the incorporation of foundational model concepts [6,7].

## 2. Evolution of EHRs in Taiwan and United States

The evolution of Electronic Health Records (EHRs) from simple digital replacements for paper charts to sophisticated systems supporting a wide range of clinical and administrative functions marks a significant advancement in healthcare technology [8–10]. The first prototype of an EHR, designed as a clinical information system, was developed by Lockheed in the mid-1960s. Around the same period, the University of Utah created an early clinical decision support system. The U.S. government began adopting EHRs in the 1970s, initially through a pilot program within the Veterans Affairs’ Decentralized Hospital Computer Program. After a decade of research, the Institute of Medicine recommended the widespread adoption of EHRs to enhance patient safety, collaborating with HL7, an international nonprofit organization, to establish standards for information exchange and operational functions within EHR systems.

**Fig. 2:**
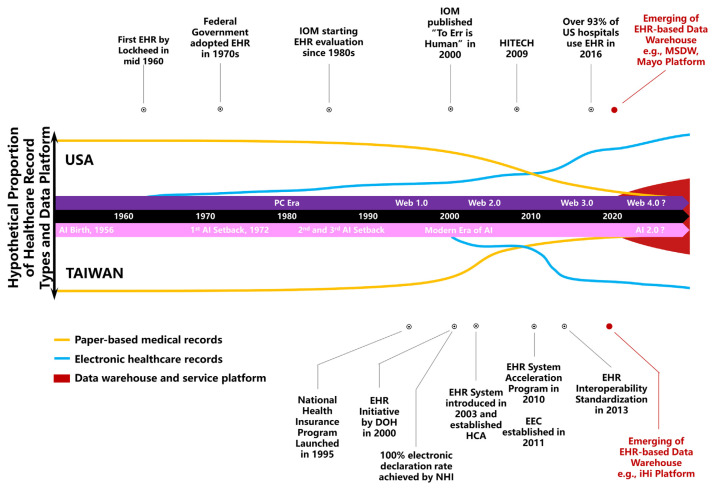
Comparative timelines and key milestones in the evolution of electronic health records (EHRs) in the United States and Taiwan, alongside the concurrent development of the internet and artificial intelligence (AI). The milestones are represented by dot circles, with the red dot indicating the emergence of data service platforms. The orange and green lines depict the trends of paper-based medical records and EHRs, respectively. The red shaded area highlights the trend toward the establishment of EHR-based data service platforms.

The adoption of EHRs in the United States gained significant momentum in 2009, when they were recognized as a key element of President Obama’s Health Information Technology for Economic and Clinical Health (HITECH) Act, which accelerated their implementation across U.S. hospitals. The digitization of healthcare rapidly expanded throughout the 2010s (Fig. 2). Although the United States led the early adoption of EHRs, the involvement of three distinct parties—government, vendors, and payers—resulted in the development of separate, non-integrated systems. As a consequence, communication between these systems remains inadequate, hindering full interoperability across various EHR platforms.

In contrast, the adoption of EHRs in Taiwan has progressed more slowly than in the United States, largely due to its close integration with the development of the National Health Insurance (NHI) system. This relationship has facilitated the creation of a robust IT infrastructure that supports interoperability across EHR systems. In 2000, following the achievement of a 100% electronic declaration rate through the NHI system, Taiwan’s Department of Health launched a nationwide EHR initiative. A pivotal milestone occurred in 2011 with the establishment of the Electronic Medical Record (EMR) Exchange Center, coordinated by the Healthcare Certification Authority Network [11], which contributed to the standardization of national EHR interoperability.

Significant progress in interoperability was made in 2016 with the implementation of the NHI Cloud-Based Inquiry System for Medical Care Information, enabling the retrieval of 12 types of clinical data, including laboratory results, surgical records, vaccination histories, and medical images from CT and MRI scans [12]. Currently, Taiwan’s Ministry of Health and Welfare is advancing an international EHR ecosystem by adopting Fast Healthcare Interoperability Resources (FHIR) [13].

The disparities in EHR development between the United States and Taiwan are multifaceted, shaped by differences in healthcare system structures and policy frameworks. In Taiwan, the level of interoperability is closely tied to reimbursement practices. For instance, hospitals experience reduced reimbursement rates if they fail to upload clinical data to the NHI Cloud. When effectively utilized, national EHR databases have the potential to serve as the foundation for developing patient digital twins (PDTs), which would document the individual disease trajectories of patients.

## 3. EHRs evolving into data service platforms in the age of AI

The unprecedented demand for AI applications in real-world settings has positioned electronic health records (EHRs) as the cornerstone of today’s health intelligence ecosystem. The primary strength of EHRs lies in their capacity to function as comprehensive repositories of patient data—encompassing demographics, medical history, vital signs, laboratory results, and imaging studies—provided that the institutional infrastructure can effectively track the patient’s data trajectory within its own EHR system [14]. When meaningfully cleaned, this rich dataset offers fertile ground for AI development, enabling algorithms to analyze disease patterns, predict patient outcomes, and support clinical decision-making [1,15].

Developing economies such as India are making substantial investments in national digital health missions, with EHRs at their core. These initiatives aim to establish interoperable systems that enhance health care delivery and improve patient outcomes [16]. Developed economies like the United States and the United Kingdom are also advancing, with data service platforms such as Stanford Data Science Resources (SDSR), Mount Sinai Data Warehouse (MSDW), and the UK’s General Practice Research Database (GPRD) facilitating large-scale, data-driven innovations in personalized medicine, advancing translational research, and supporting intelligent clinical decision-making [17–19]. The future potential of data intelligence can be maximized when EHRs are further integrated with national biobanks based on general populations, such as the All of Us initiative, the UK Biobank, and the Taiwan Biobank [20–22].

Similar to current pharmacological standards, where drug approvals rely on multicenter clinical trials to ensure generalizability, AI- and data-driven digital solutions must undergo a comparable clearance process [23]. A critical challenge in this context is the fragmentation of data within and across healthcare systems [24], further complicated by legacy issues and incompatible information technology infrastructures [25]. Overcoming these challenges is essential to fully harness data intelligence and AI in medicine and public health. Another major challenge involves heterogeneous data quality, stemming from inconsistent data governance strategies and variations in common data models, such as the Observational Medical Outcomes Partnership, as well as issues related to missing values or incomplete data entries due to regional practice differences and inadequate follow-up [26,27].

Despite growing global interest in AI applications, the medical field continues to face delays in data preparation. Increasing awareness underscores that data is the essential fuel required to unlock AI’s potential, from disease prognosis prediction to liability control, positioning EHRs at the core of the clinical AI ecosystem.

## 4. Data services in leading research platforms

The adoption of data services by prominent U.S. institutions accelerated in the 2010s, with leaders like the Mayo Clinic, Stanford University, and Mount Sinai Medical Center integrating electronic health records (EHRs) as primary repositories for translational research [17,18,28]. This shift paralleled the rapid advancement of artificial intelligence (AI) technologies, yet the emphasis on AI applications sometimes overshadowed the essential role of foundational data intelligence needed to drive meaningful AI insights. Each platform represents unique data governance strategies aimed at reducing data silos and enhancing data quality. This article offers a concise overview of the core characteristics of major platforms, including the Mayo Clinic Platform, the Mount Sinai Data Warehouse (MSDW), the Stanford Data Science Resources (SDSR), and the General Practice Research Database/Clinical Practice Research Datalink (GPRD/CPRD).

### 4.1. Mayo Clinic Platform

The Mayo Clinic Platform integrates data from over 10 million patients, comprising 10.4 million records. This comprehensive dataset includes 1.6 billion laboratory test results, 10 million pathology reports, 698 million clinical notes, and more than 400 million medical images. Among the images are 241 million CT scans, 146 million MRI scans, 10.2 million electrocardiograms, and 2.5 million positron emission tomography scans [28]. All clinical data are de-identified in accordance with Health Insurance Portability and Accountability Act (HIPAA) regulations, ensuring that both structured and unstructured information is anonymized to protect patient privacy [29].

Built on this secure infrastructure, Mayo Clinic, in collaboration with Google, has developed a cloud-based Clinical Data Analytics Platform to enable secure data sharing. This platform allows third parties to develop and validate novel algorithms and conduct data analyses while ensuring that data remains securely within Mayo Clinic’s environment, never leaving the Mayo container [30]. This approach has unlocked the commercial potential of the Mayo Clinic Platform, leading to the launch of the Mayo Accelerate Program, which partners with global startups to test and validate their AI-driven or data intelligence-based digital solutions.

### 4.2. Mount Sinai Data Warehouse (MSDW)

MSDW collects clinical and operational data from the Epic Electronic Health Record (EHR) system at Mount Sinai Health System, granting researchers access to over 11 million patient records and more than 87 million patient encounters since its inception in 2011 [18]. The Mount Sinai Health System, which includes facilities such as Mount Sinai Hospital, Queens, West, Morningside, Brooklyn, and Beth Israel, transitioned to the Epic EHR system between 2011 and 2020. Along with other ancillary systems, Epic serves as the primary data source for MSDW. To facilitate optimal data sharing and interoperability both internally and externally, clinical data are extracted and transformed into the Observational Medical Outcomes Partnership (OMOP) common data model. The dataset is updated daily, ensuring the availability of current data for research and operational purposes.

MSDW operates on the Minerva High-Performance Computing (HPC) cluster, which also supports other research datasets [31]. Minerva has been HIPAA-compliant since October 1, 2020, ensuring the secure storage and processing of protected health information.

### 4.3. Stanford Data Science Resources (SDSR)

The Stanford Medicine Data Science Resource (SDSR) provides access to large-scale datasets through key repositories, including the Stanford Medicine Research Data Repository (STARR), the Stanford Cancer Institute Research DataBase (SCIRDB), and the Population Health Sciences (PHS) Data Portal. STARR contains over two decades of patient data from Stanford Health Care and Stanford Children’s Health, with updates occurring every 24–36 h [17]. SCIRDB integrates cancer research data with national health insurance registries, while the PHS Data Portal hosts 83 population health datasets [32], making these resources essential for advancing health science research [33].

The SDSR data science ecosystem is underpinned by a HIPAA-compliant computing infrastructure that supports secure search, access, analysis, and de-identification pipelines, enabling the extraction of valuable insights from healthcare IT systems [17]. Notably, Stanford’s PHS data ecosystem was specifically developed to securely manage and share large-scale, high-risk health data covering hundreds of millions of individuals. It is accessible to researchers at Stanford University and is designed with replication capabilities for use at other institutions.

Despite the platform’s effectiveness in hosting and curating vast datasets, challenges persist in enhancing data discoverability, accessibility, and reusability, which are critical for fully supporting translational research [32]. To ensure long-term support for translational research using real-world data, the implementation of advanced technological solutions, robust management structures, and educational initiatives that foster collaboration among researchers, data scientists, and the broader community have been identified as key requirements [32].

### 4.4. General Practice Research Database/Clinical Practice Research Datalink (GPRD/CPRD)

The General Practice Research Database (GPRD), renamed the Clinical Practice Research Datalink (CPRD) in 2012, is a computerized repository containing anonymized patient records [19]. Since its inception in 1987, the CPRD has continuously accumulated data and currently holds information on 60 million patients from the United Kingdom, including 18 million actively registered patients [34]. The CPRD provides real-world data services that support both retrospective and prospective public health and clinical research. These services are managed by the Medicines and Healthcare Products Regulatory Agency (MHRA) with support from the National Institute for Health and Care Research (NIHR) [35,36].

The CPRD encompasses anonymized electronic health records (EHRs) from primary care practices, including patient demographics, diagnoses, medication exposures, and laboratory test results. Additionally, it integrates primary care data with other health datasets, such as hospital admissions, mortality records, cancer registries, and socioeconomic data, providing a comprehensive perspective on patient health. To maintain public trust and ensure data integrity, the CPRD has established the Research Data Governance process, which enables secure access to its data while upholding legal and ethical standards for research and healthcare purposes [37].

As institutions develop their own artificial intelligence (AI) ecosystems, driven by advanced data service platforms, a key challenge lies in data governance. One major issue is the approach to data cleaning: many medical centers adopt a decentralized model, where datasets are made available through service platforms or access-controlled tables, allowing users to work with raw data. In this model, users are often responsible for developing their own data cleaning methods to ensure data quality before analysis. Alternatively, a centralized data cleaning approach, supported by standardized codebooks, provides a more efficient solution. This method organizes high-quality, theme-based databases, enabling users to access clean and well-structured data. Furthermore, centralized cleaning facilitates the integration of multiple high-quality datasets, supporting the “data LEGO” concept, ensuring analytical accuracy, and mitigating the “garbage in, garbage out” problem.

### 4.5. Insufficient integration of external clinical information

One of the key challenges in health-care data management is the inadequate integration of clinical information from external sources [38]. Patient data are often dispersed across various hospitals, insurance providers, and government agencies, leading to data fragmentation, which can introduce bias into hypothesis testing and reduce the efficiency of AI model development. Data ecosystems developed by insurance payers, such as Kaiser Permanente and Geisinger, are often better integrated because they encompass data from multiple hospitals within a unified, insurance based healthcare system [39,40]. However, when patients seek care from different healthcare systems or change insurance providers, linking their medical data remains a challenge. In Taiwan, the universal healthcare system presents a unique opportunity to develop a comprehensive patient tracking environment with minimal loss to follow-up [41].

### 4.6. Limited scope of EHRs in PDTs

Electronic Health Records (EHRs) capture approximately 20% of a patient’s total life data, which is inadequate for constructing a comprehensive Patient Digital Twin (PDT)—a virtual representation of an individual’s health trajectory [6]. Even with the integration of genomic and other omics data, this percentage increases only marginally, reaching about 30% [7]. The majority of relevant patient data—estimated at 70%–80%—arises from daily activities such as lifestyle factors (e.g., diet, sleep, exercise) and environmental exposures. However, these factors are typically not included in traditional EHRs. This discrepancy highlights the need for standardization in incorporating data from Internet of Things (IoT) devices and app-generated health information into healthcare systems, potentially through standardized data formats like FHIR [42]. Therefore, the development of next-generation EHR systems is urgent. These systems would not only serve as foundational components for PDT construction but also facilitate the seamless integration of artificial intelligence (AI) technologies. As AI applications increasingly rely on diverse and comprehensive datasets, addressing this gap is critical for advancing personalized medicine and intelligent healthcare—a challenge that extends beyond the scope of this review.

In summary, Electronic Health Records (EHRs) have gradually transformed into advanced data service platforms that support modern clinical decision support systems incorporating artificial intelligence (AI) and data analytics since the mid-2010s. To further enhance the integration of AI in healthcare, the next critical step will be the unification of these service platforms across diverse economic and healthcare systems.

## 5. Overview of the iHi data platform

**Fig. 3:**
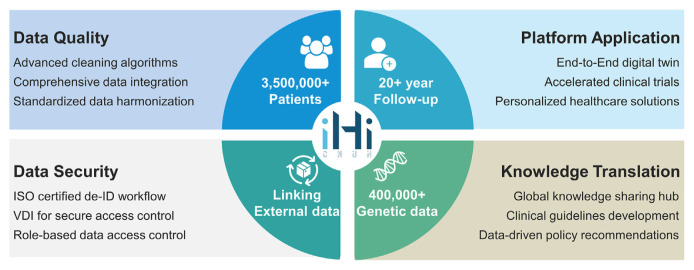
Overview of the iHi Platform: Data Quality, Security, Application, and Knowledge Translation. VDI refers to Virtual Desktop Infrastructure.

China Medical University Hospital (CMUH), the largest medical center in central Taiwan, has been dedicated to establishing a robust medical data ecosystem to support AI and digital infrastructure for a smart health-care system [43]. Since 2017, the Big Data Center (BDC) at CMUH has led the development of the iHi Platform, a comprehensive data ecosystem aimed at fostering hyper-intelligence in health care. This large-scale, interoperable platform integrates phenotypic, genomic, and environmental data, including 20 years of electronic medical records (EMRs) and environmental exposure data from over 3.5 million patients, along with genetic data from 400,000 individuals. Additionally, the platform is linked with National Health Insurance (NHI) data (Fig. 3).

The iHi Platform operates with a “data LEGO” approach, featuring a standardized data cleaning pipeline, integrated infrastructure, an ISO-certified, regulation-compliant de-identification process, and a secure virtual working environment. These components enable web-based data exploration and support no-code multi-omics analysis, offering extensive and integrated real-world evidence. This infrastructure not only enhances the quality of medical education and supports clinical research but also promotes the sustainability of healthcare innovations.

### 5.1. iHi platform infrastructure for comprehensive data services

**Fig. 4:**
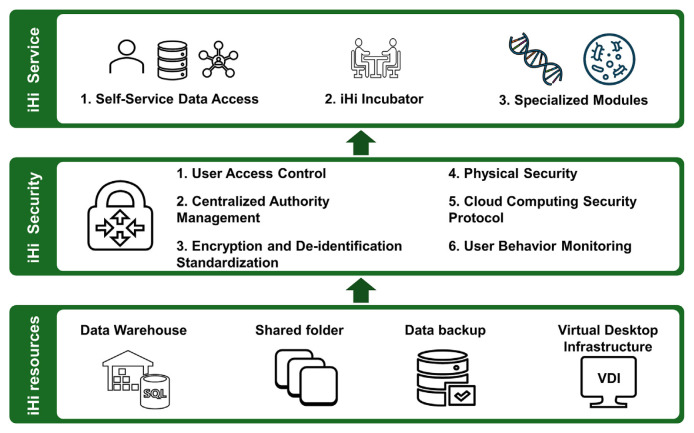
Infrastructure and Data Workflow for the iHi Platform’s Data Services.

The iHi platform provides comprehensive, secure data services, emphasizing both data protection and user accessibility (Fig. 4). To meet diverse user needs, the platform offers three distinct service models. For users with data analysis expertise, iHi provides access to fully cleaned, de-identified datasets, with an option to incorporate external data. For users with research concepts but limited analytical skills, the iHi Incubator enables collaboration with data scientists from CMUH’s BDC to develop and complete research projects. Additionally, the platform includes specialized modules for genetic and microbiome analyses, allowing researchers to link phenotype, genotype, and microbiome data for advanced studies.

All services are conducted within a secure, personalized workspace through a Virtual Desktop Infrastructure (VDI), ensuring that data remain within the iHi environment. This setup allows users to securely access and analyze data remotely. All data are sourced exclusively from individuals who have provided consent, and CMUH has implemented a real-time consent revocation feature since 2022 via its patient service app, CARES.

### 5.2. Data de-identification

**Fig. 5:**
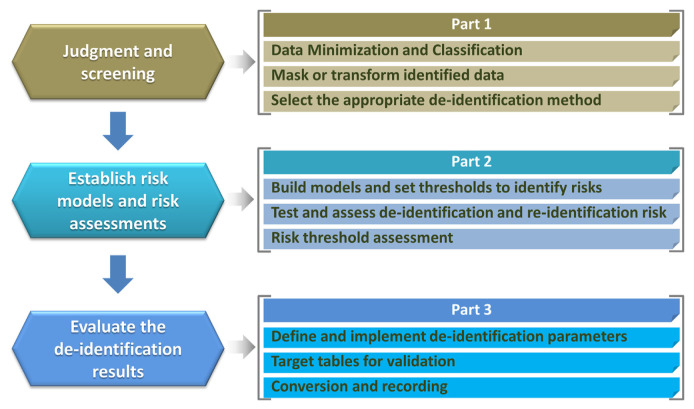
ISO-Certified De-identification Workflow for the iHi Database.

CMUH is the first hospital in Taiwan to achieve both domestic and international dual certifications for its clinical data and image database, meeting the ISO 29100 and 29191 standards as well as CNS 29191 and CNS 29100-2 standards. The iHi platform utilizes the BDC’s automated de-identification system (Fig. 5) to ensure strong data security while supporting diverse data applications. This system applies various automated de-identification techniques, including generalization, suppression, K-anonymity, and image masking, to protect sensitive health information [44]. After de-identification, a comprehensive risk assessment is performed, followed by consistency checks and public database searches to confirm that no personally identifiable information remains.

To further enhance privacy protections, the platform continuously adopts new technologies to address emerging data types, such as those generated by wearable devices like smartwatches and fitness trackers. Additionally, CMUH is actively pursuing additional international certifications, including ISO/ IEC 20889:2018 (Privacy-Enhancing Data De-identification Terminology and Classification of Techniques) and ISO/IEC 27559:2022 (Information Security, Cybersecurity, and Privacy Protection - Privacy-Enhancing Data De-identification Framework).

### 5.3. Data cleaning and harmonization: smart data chip workflow

**Fig. 6:**
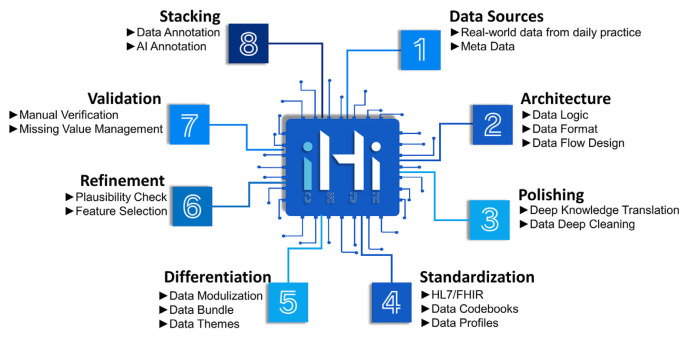
Smart data chip workflow for data cleaning and harmonization.

Data derived from routine clinical practice often contain errors and inconsistencies, making data quality—encompassing completeness, consistency, and validity—critical for constructing accurate patient digital twins (PDTs) and supporting AI-driven healthcare solutions [45]. To address these challenges, we propose a comprehensive data cleaning and harmonization framework known as the Smart Data Chip Workflow (Fig. 6). This workflow underpins the iHi platform’s database through systematic quality control processes, transforming raw clinical data into high-quality, research-ready datasets.

The workflow begins by integrating clinical data from multiple sources to break down data silos. These data are structured within a well-defined architecture, ensuring logical consistency and proper formatting, thereby facilitating seamless integration across institutions. Domain experts, alongside various data-cleaning methodologies (e.g., outlier detection and unit consistency checks), refine the data to ensure accurate terminology mapping and the resolution of inconsistencies. To further enhance interoperability, clinical data ontologists label and classify the data, ensuring alignment with multiple formats and international frameworks, such as HL7 and FHIR.

Next, the data are organized into specific themes and modules, followed by rigorous plausibility validation to ensure consistency with clinical practices and medical standards. A series of statistical analyses and validation steps (e.g., descriptive statistics) are then conducted to verify data accuracy and reliability. Each step in this process is meticulously documented, generating a comprehensive data lineage that tracks the entire history of data processing, thereby ensuring transparency and reproducibility.

**Fig. 7:**
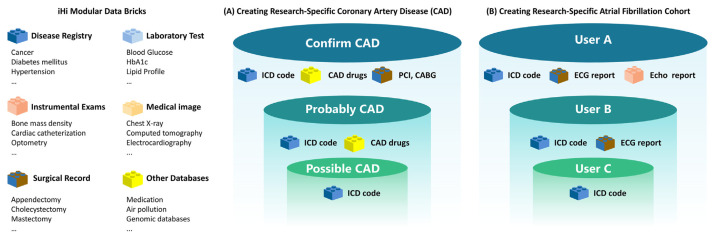
iHi Platform Data Organization: Modular Data Bricks to Enhance Research Flexibility and Interoperability. Panel (A) presents a multi-level coronary artery disease (CAD) cohort, categorized into “Confirmed CAD,” “Probable CAD,” and “Possible CAD” groups, each using different combinations of data modules such as ICD codes, CAD medications, percutaneous coronary intervention (PCI), and coronary artery bypass grafting (CABG). Panel (B) illustrates a customizable Atrial Fibrillation cohort, where User A, User B, and User C build datasets by selecting modules such as ICD codes, electrocardiogram (ECG) reports, and echocardiogram (Echo) reports according to their specific research needs. This modular design supports flexible, personalized data configurations for a variety of research applications. CABG, coronary artery bypass grafting; CAD, coronary artery disease; ECG, electrocardiogram; PCI, percutaneous coronary intervention.

The final product, developed through the Smart Data Chip Pipeline, ensures data interoperability and expandability, akin to LEGO bricks. This allows for customization to meet specific user or client needs (Fig. 7). Users can select and combine data modules to construct research-specific databases, supporting advanced analytics, AI model development, and a wide range of clinical and research applications.

## 6. Integrated multiomics analysis platforms: genomics and microbiome

### 6.1. Overview of iHi genomics

While many countries have established biobanks [46], genetic research often demands specialized analytical expertise, substantial computational resources, and extensive data storage to handle large-scale genomic datasets. To address these challenges and improve data accessibility within CMUH, the iHi Platform developed iHi Genomics, an automated genetic analysis platform. This platform integrates genetic data from approximately 400,000 individuals, complemented by 19 years of longitudinal clinical data.

iHi Genomics consolidates CMUH’s institutional infrastructure into a high-performance computing environment, featuring 128 processing threads, 2 TB of RAM, and 1.2 PB of storage capacity. Through optimized task scheduling and minimizing idle resource time, the platform maximizes both performance and efficiency. This enables users to perform genetic research with no programming requirements, streamlining the process and enhancing accessibility for researchers across disciplines.

### 6.2. Precision Medicine Project

The CMUH Precision Medicine Project (PMP), initiated in 2018, has continuously enrolled outpatient participants in a sequential manner, with all participants providing written informed consent [47]. The PMP collects genetic data using the TWBv2 genotyping array, a Taiwanese-specific custom SNP array based on the ThermoFisher Axiom platform. This array incorporates rare coding risk alleles derived from whole-genome sequencing data of Taiwanese samples [48]. Specifically, the TWBv2 array includes 114,000 risk variants spanning 2831 rare disease genes identified in the published literature and the ClinVar database, as well as 4100 variants related to drug metabolism and adverse drug reactions. Additionally, the array features 24,865 copy number variation (CNV) probes associated with known chromosomal aberrations and CNV regions [22].

**Fig. 8:**
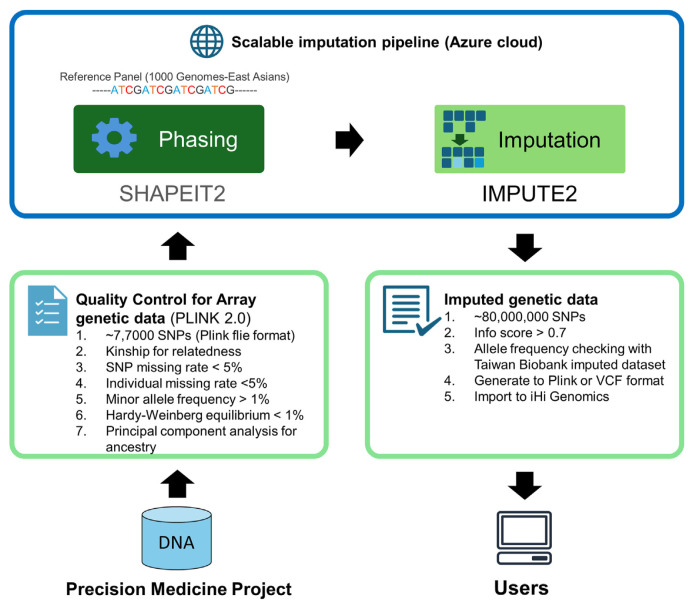
A scalable genotype imputation pipeline through a hybridized cloud and local system. Genotype imputation is performed using SHAPET2 and IMPUTE2 tools. The pipeline integrates a cloud-based platform for scalable computational power and a local data platform to ensure data security and privacy.

All genetic data in the iHi Genomic platform undergo rigorous quality control assessments, evaluating factors such as kinship, ancestry, missing data rates, allele frequencies, and adherence to Hardy–Weinberg equilibrium [49]. To examine gene-clinical outcome associations on a whole-genome scale, missing genotypes were imputed using the 1000 Genomes Project phase 3 East Asian panel as a reference [50]. Haplotype phasing was performed with SHAPEIT2, and imputation was conducted using IMPUTE2 [51,52], generating a total of 81,698,455 imputed SNPs. We retained imputed SNPs with an INFO score >0.7, yielding a final dataset of 22,537,475 imputed SNPs (Fig. 8). All quality control procedures were conducted using PLINKv2.0.19.

As of 2024, the iHi Genomics cohort consists of 400,458 samples, with a median age of 46.3 years (IQR=30.1–62.3), and a male representation of 45.8%.

### 6.3. Standardized analytic pipelines for genome-wide association studies (GWAS) and mendelian randomization

**Fig. 9:**
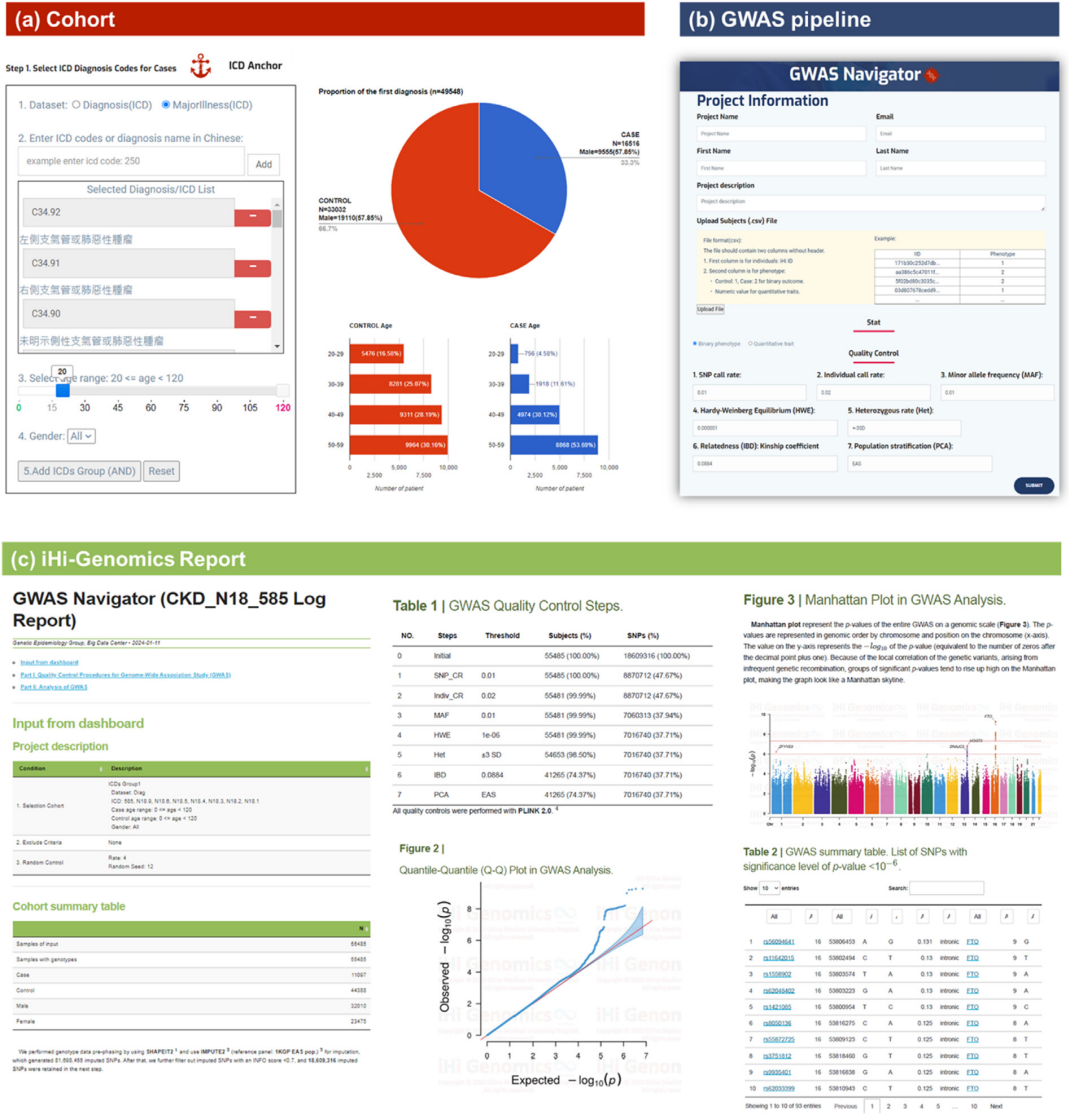
User-friendly interface of iHi-Genomics. The iHi-Genomics platform consists of two functional modules: ICD Anchor (a) and GWAS Navigator (b). Each module features an intuitive user interface, enabling users to perform genome-wide association studies (GWAS) with no programming expertise required. The platform generates a comprehensive GWAS report (c), which includes detailed information on each quality control step, a Q–Q plot, a Manhattan plot, and an interactive SNP list. This well-organized report offers researchers a clear and informative overview of their GWAS analysis.

In 2022, we launched iHi Genomics, an automated platform designed to streamline Genome-Wide Association Study (GWAS) analyses. This platform facilitates the establishment of target cohorts by using the International Classification of Diseases (ICD) coding system and enables users to control the quality of genetic data, perform zero-programming analyses, and summarize GWAS results comprehensively through visualizations and interactive tables. By using the ICD Anchor interactive dashboard, users can select cases based on ICD codes for specific diseases, with the system automatically matching appropriate controls and providing visualizations of demographic data for both cases and controls. Once the study cohort is confirmed, the platform retrieves the corresponding genetic data from iHi Genomics, processes it through stringent quality control protocols, and proceeds with GWAS analysis using the GWAS Navigator module. Within 24 h, users receive a comprehensive GWAS report that contains details of each quality control step, a Quantile–Quantile (QQ) plot, a Manhattan plot, and an interactive annotation table of statistically significant SNPs (Fig. 9). This table enables users to filter results by rsID number, p-value, chromosome position, and gene symbols, and includes links to external public databases such as GeneCards for streamlined access to gene-related information [53].

Another application of genetic data is Mendelian randomization (MR), frequently used to evaluate the causal relationships between exposures and outcomes [54], especially when randomizing exposures would be ethically or practically infeasible [55]. MR leverages genetic variants as instrumental variables to simulate randomization trials and relies on three key assumptions [56]. First, the genetic instruments must be strongly associated with the exposure. Second, these instruments should not be associated with confounding variables. Third, no horizontal pleiotropy should exist, meaning that genetic instruments influence the outcome solely through the exposure. We adopted the “TwoSampleMR” R package to establish an MR pipeline, which supports a two-sample MR study and validates the critical assumptions of the MR model [57]. For example, users can assess the association between the genetic instruments and exposure by using F-statistics [F = (R^2^(N – K – 1))/(1 – R^2^)K, where N is the sample size, K is the number of genetic variants in the instrument, and R^2^ is the percentage of variance in the exposure explained by the genetic instrument [56]. Typically, F-statistics >10 suggest a strong association between instruments and exposure [58]. To address potential horizontal pleiotropy, we employed MR-Egger regression, where a nonsignificant intercept value indicates no evidence of pleiotropy affecting the outcomes [59]. Furthermore, sensitivity analyses were performed using various MR methods, including inverse-variance weighted (MR-IVW), MREgger regression, weighted median (MR-median), weighted mode (MR-mode), simple-mode, and Mendelian Randomization Pleiotropy RESidual Sum and Outlier (MR-PRESSO) [60]. Although our MR services are currently customized according to specific applicant needs, a dedicated digital platform is under development.

### 6.4. Overview of iHi microbiome

Microbial marker genes, particularly 16S rRNA, are essential for microbiome profiling, offering rapid and reliable insights into microbial communities [61]. However, current computational tools for microbiome analysis often require users to have programming expertise for tool integration and statistical method selection, presenting a barrier for researchers without coding experience [62]. To address this challenge, we developed iHi Microbiome, a comprehensive, flexible, and user-friendly web-based platform for microbiome analysis. This platform integrates a wide array of functionalities, including sequence data processing and advanced analysis, to meet the needs of both novice and expert researchers.

**Fig. 10:**
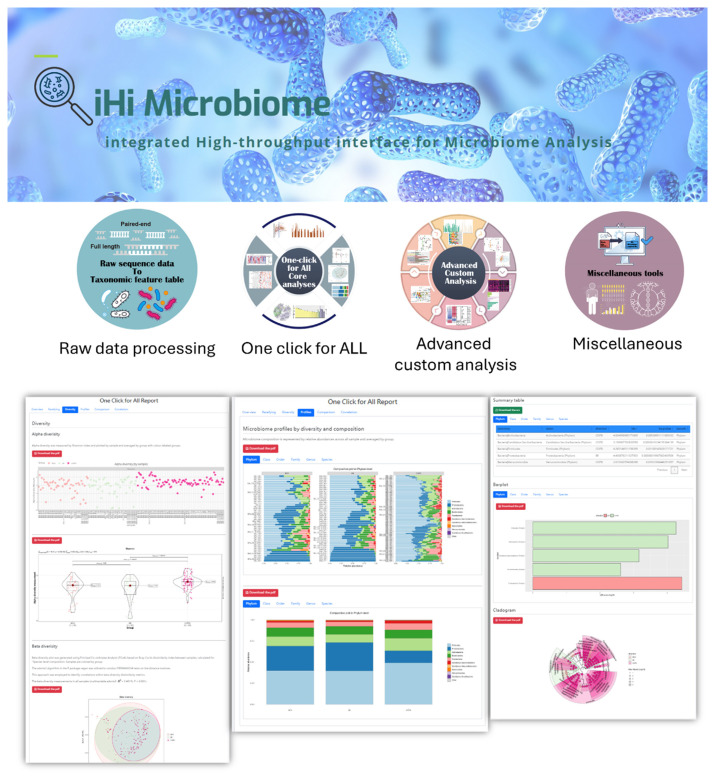
The iHi-Microbiome platform offers comprehensive functionality in four main areas: (1) Raw Sequence Data Processing, ensuring robust data analysis, quality control, and accurate taxonomic assignments; (2) One-click-for-all Analysis, providing a user-friendly interface for diverse analyses, including Alpha- and Beta-diversity, microbiome composition, differential abundance, and correlation analysis; and (3) Advanced Custom Analysis, tailored to specific study requirements. The One-click-for-all Analysis includes Alpha-diversity to assess richness and evenness within samples, Beta-diversity to measure community variability across samples, and microbiome composition profiling across different habitats. The summary table also includes a cladogram displaying differentially abundant taxa across habitats, thereby enhancing comparative insights across sample groups.

The iHi Microbiome platform offers four core functions: (1) raw sequence data processing, (2) a one-click-for-all function, (3) advanced custom analysis, and (4) miscellaneous tools (Fig. 10). The raw sequence data processing feature enables robust data analysis, quality reporting, and accurate taxonomic assignment. The one-click-for-all function provides a simplified, user-friendly solution for various microbiome analyses.

Users can perform complex analyses, such as microbial community biodiversity, microbiome composition, differential abundance, and correlation analyses, by selecting appropriate methods and adjusting parameters. The advanced custom analysis and miscellaneous tools offer more sophisticated, customizable options with detailed parameter settings, addressing specific research requirements, including functional prediction, pathway analysis, and a range of statistical models and data visualization tools. iHi Microbiome thus serves as an efficient, comprehensive, and adaptable tool for microbiome research. It is a free, open-access platform, requiring no login, ensuring broad accessibility. The platform is available at https://cmuh-ihi-microbiome.nchc.org.tw/. In comparison to other microbiome analysis platforms, such as MicrobiomeAnalyst and MOCHI, which offer a wide array of tools for microbiome data analysis, the iHi Microbiome platform is distinguished by its seamless integration within the iHi data ecosystem. This integrated approach allows users to link microbiome data with clinical, genomic, and environmental datasets, thereby facilitating comprehensive, multi-dimensional analyses. Such functionality provides an expanded perspective for microbiome research, enabling deeper insights into the complex interactions between microbiome composition and various health determinants.

### 6.5. User interface design

The iHi Genomics and iHi Microbiome platforms are designed as streamlined, zero-programming data analytics tools aimed at reducing the learning curve for clinical researchers without bioinformatics expertise. Both platforms emphasize user-friendly, web-based dashboards that simplify navigation through complex datasets, enabling users to derive meaningful insights through intuitive operations and visually engaging data presentations. These platforms feature guided graphical user interfaces (GUIs), ensuring that all tasks can be performed without the need for coding knowledge.

### 6.6. Future perspectives

iHi Genomics represents Taiwan’s first large-scale platform seamlessly integrating genomic and phenotypic data. This integration holds significant potential for advancing precise disease risk assessments and predictions, guiding new drug development, and promoting innovation in clinical practice. By further connecting to environmental databases, our unique and robust data ecosystem enables researchers to comprehensively examine the interactions between genetic, environmental, and clinical factors in disease progression.

While this is an initial step toward the era of big data and AI, the iHi Platform, encompassing iHi Genomics and iHi Microbiome, provides a solid foundation for developing comprehensive patient digital twins (PDTs). This potential is amplified as diverse omics data, including transcriptomics, proteomics, and single-cell RNA sequencing, become integrated. Moreover, with the application of advanced IoT technologies, the platform could connect to real-time daily activities, creating a dynamic, Avatar-like representation of patients.

Strategically, the iHi Digital Data Ecosystem is poised to integrate with future metaverse applications, enabling seamless, real-time interactions between digital avatars of patients, healthcare professionals, and their living environments.

## 7. Discussion and conclusion

The data ecosystem established by the iHi Platform forms the foundation for the development of patient digital twins (PDTs) through the integration of data from National Health Insurance (NHI) records, multi-omics profiles, and environmental exposures. This integration is further enhanced by disease management apps, which link the ecosystem to real-time data generated by patients, enabling comprehensive profiling of patients’ daily activities. Despite these advancements in data governance and quality, critical gaps remain, including the lack of dynamic integration with patients’ living environments and the absence of interconnected digital twins representing other healthcare professionals and patients. These missing elements are essential for fully leveraging the potential of cutting-edge AI technologies, such as generative AI and large language models.

The vision of a Global Patient Digital Twin Initiative (GPDTI) presents the challenge of international interoperability. Unifying diverse clinical data service platforms across various healthcare systems and hospitals could revolutionize the efficiency of AI solution validation and enable the creation of digital twin-driven virtual clinical trials. However, despite HL7’s efforts to promote the global adoption of FHIR, the integration of this cross-institution communication protocol has been slow [63]. The current standards development process, led by HL7, focuses on adapting to new data types, including mobile app data, patient audio and video recordings, and advanced multi-omics data, such as microbiome and proteomics information [64,65]. However, data standards governing AI-generated biomedical data, including interactions between generative AI and patients or clinical professionals, AI-driven disease prognosis predictions, and AI-simulated videos (e.g., gait analysis), remain underdeveloped and lack universal agreement. Given the variability of AI tool accuracy across healthcare institutions, implementing standardization mechanisms prior to integrating these inferences is essential. This may involve retraining AI models using all available data or applying bias-correction approaches to these inferences. A thorough evaluation of standardization strategies is necessary before deploying data standardization protocols for AI inferences [66]. Despite these challenges, AI-derived digital biomarkers or biosignatures are critical for realizing digital twins, which are pivotal for applications such as virtual clinical trials and disease trajectory simulations within the next generation of Health Information Systems (HIS)/Electronic Health Records (EHRs).

A key element of the GPDTI is the implementation of comprehensive data collection strategies that strictly adhere to privacy-preserving practices. This poses a significant challenge for the next generation of EHR systems, which require international interoperability while complying with national and regional patient privacy laws, such as HIPAA in the United States and the General Data Protection Regulation (GDPR) in the European Union. At CMUH, the BDC adheres to regulations like Taiwan’s Personal Information Protection Act, ensuring patient confidentiality while enabling data-driven healthcare innovations through a careful balance of access and protection. Furthermore, the export of clinical data, particularly genetic data, is tightly regulated by the Taiwanese government. Therefore, the principle of “data never leaving” serves as a fundamental guideline. The iHi Platform employs Virtual Desktop Infrastructure (VDI) services to uphold this policy in international AI research collaborations, complemented by rigorous data anonymization and deidentification techniques using AI methods. However, the “data never leaving” policy, which restricts data to physical locations within Taiwan, may hinder the progress of the GPDTI, as the exchange of data at the digital twin level—especially multi-omics data—is essential for meaningful collaboration. To address this, the development of an intelligent consent management system is crucial, ensuring that patients are fully informed about the scope and purpose of data sharing with third parties, including research institutions and commercial entities. Importantly, patients must have the ability to revoke consent at any time for specific types of information, in line with GDPR principles. Additionally, all data service platforms, including iHi, should adopt stringent access controls based on the principle of least privilege, implement real-time monitoring, and maintain detailed logs of data access. Despite these safeguards, the potential for irreversible damage to patient rights and organizational reputation in the event of a data breach remains a serious concern that cannot be fully mitigated through legal action. Establishing a secure yet open data ecosystem to support the integration of AI in clinical practice will require substantial legal and regulatory efforts, especially in an era of rapid advancements in data science and AI technology.

As AI-driven systems become integral to healthcare, effective error management is essential for establishing a framework of Trustworthy AI and fostering digital trust among clinicians and patients. While AI significantly enhances diagnostic and therapeutic decision-making, it also presents potential risks associated with errors and biases originating from training data. To mitigate these risks, explainable AI tools [67], such as SHAP, play a critical role by providing transparency into model decision-making processes, thereby enhancing model reliability and enabling healthcare professionals to interpret the AI’s rationale. Furthermore, practice-aligned calibration (PAC) methods can help AI models adapt to varying clinical contexts [68], promoting consistent performance and reinforcing trust in the accuracy and safety of AI applications. Together, these strategies contribute to a secure, transparent, and ethical framework for AI-driven healthcare, which is essential as the iHi Platform advances toward broader applications in clinical practice.

In conclusion, the iHi Platform represents a significant milestone in Taiwan’s evolution of medical big data by transforming conventional EHRs into a sustainable, infinite data ecosystem. Supported by government and regulatory bodies, the iHi platform is poised to drive innovation beyond the traditional learning health system within local healthcare institutions. More broadly, it aims to establish a comprehensive data ecosystem that connects to national and global policymaking, drives transformation in the international healthcare industry, and supports robust academic applications across both basic and applied sciences, including AI in medicine and public health.
